# Eviscération transanale de l´intestin grêle par empalement chez l´enfant: à propos d´un cas

**DOI:** 10.11604/pamj.2020.37.320.18332

**Published:** 2020-12-07

**Authors:** Ibrahima Bocar Wellé, Pape Alassane Mbaye, Ndéye Fatou Séck, Ndéye Aby Ndoye, Doudou Guéye, Faty Balla Lo, Modou Ndiaye, Mouhamed Aly Sylla, Aloise Sagna, Gabriel Ngom

**Affiliations:** 1 Service de Chirurgie Pédiatrique, Hôpital d'Enfant Albert Royer, Sénégal,; 2 Service de Chirurgie Générale, Hôpital de Ourossogui, Sénégal

**Keywords:** Eviscération transanale, intestin grêle, empalement, enfant, Sénégal, Transanal Evisceration, small bowel, impalement, child, Senegal

## Abstract

L'éviscération transanale de l'intestin grêle par empalement est exceptionnelle chez l'enfant. Nous rapportons l'observation d'un adolescent de 11 ans reçu à 2 heures d'une issue des anses grêles à travers l'anus suite à une chute avec réception sur un morceau de bois pointu. L'examen clinique avait objectivé un bon état général, une issue, par l'anus, d'environ 25 cm d'intestin grêle viable et un abdomen sensible dans son ensemble. Le bilan biologique préopératoire était normal et aucune imagerie n'était réalisée. Après une réanimation, l'exploration chirurgicale avait mis en évidence un liquide séro-hématique (300 ml) et une issue d'environ 60 cm d'anse grêle inflammatoire à travers une brèche d'environ 5 cm de la paroi antérieure du rectum. Une réduction des anses grêles éviscérées par traction douce, une réparation de la paroi rectale par des points séparés, une toilette et un drainage ont été réalisés. Le patient était sous antibiotique à large spectre. Les suites opératoires étaient simples avec une reprise du transit à J2 post-opératoire. La sortie a été autorisée à J7 post opératoire. Après un recul d'un mois, la patiente a été revue en consultation et l'examen clinique était normal.

## Abstract (en)

Transanal evisceration of the small intestine caused by impalement is exceptional among children. We report the case of an 11-year old girl with intestinal loops eviscerating through the anus due to a fall on a sharp piece of wood which stuck into her, occurring two hours before hospitalization. On examination, his general condition was good, with transanal evisceration of approximately 25 cm of viable small bowel through the anus and abdominal tenderness. Preoperative laboratory tests were normal and no imaging test was performed. After resuscitation, surgical exploration was performed which showed serohematic fluid collection (300 ml) and evisceration of approximately 60 cm of inflammated intestinal loops through a rupture of approximately 5 cm of the anterior wall of the rectum. Reduction of the eviscerated intestinal loops by gentle traction, repair of the rectal wall by separated sutures, wash and drainage were performed. The patient received broad-spectrum antibiotic. The operating suites were simple with a resumption of transit two days after surgery. The patient was discharged seven days after surgery. After a follow-up period of one month, the patient came for a further consultation and clinical examination was normal.

## Introduction

L'éviscération intestinale est plus retrouvée dans les plaies abdominales qu'ano-rectales et le plus souvent chez l'adulte que chez l'enfant. Ainsi l'éviscération transanale de l'intestin grêle par empalement chez l'enfant constitue un fait rare. Nous rappelons un cas d'empalement avec éviscération transanale chez un enfant en discutant les aspects épidémiologiques, diagnostiques, thérapeutiques et évolutifs.

## Patient et observation

**Figure 1:**
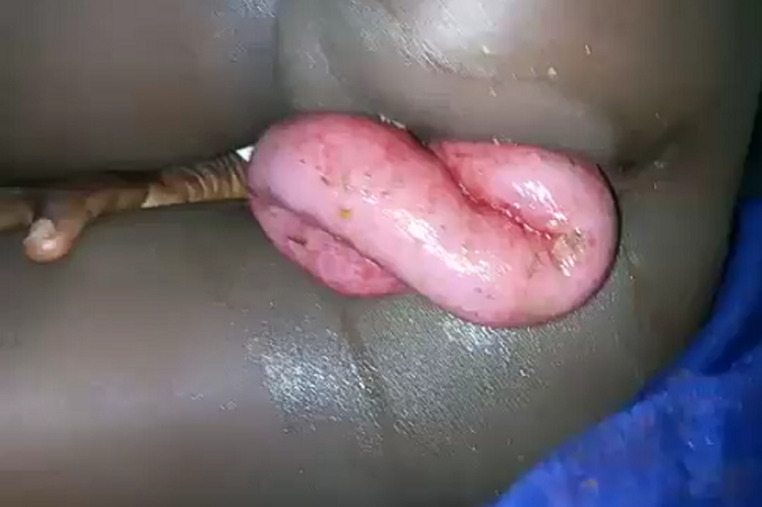
éviscération transanale de l’intestin grêle

**Figure 2:**
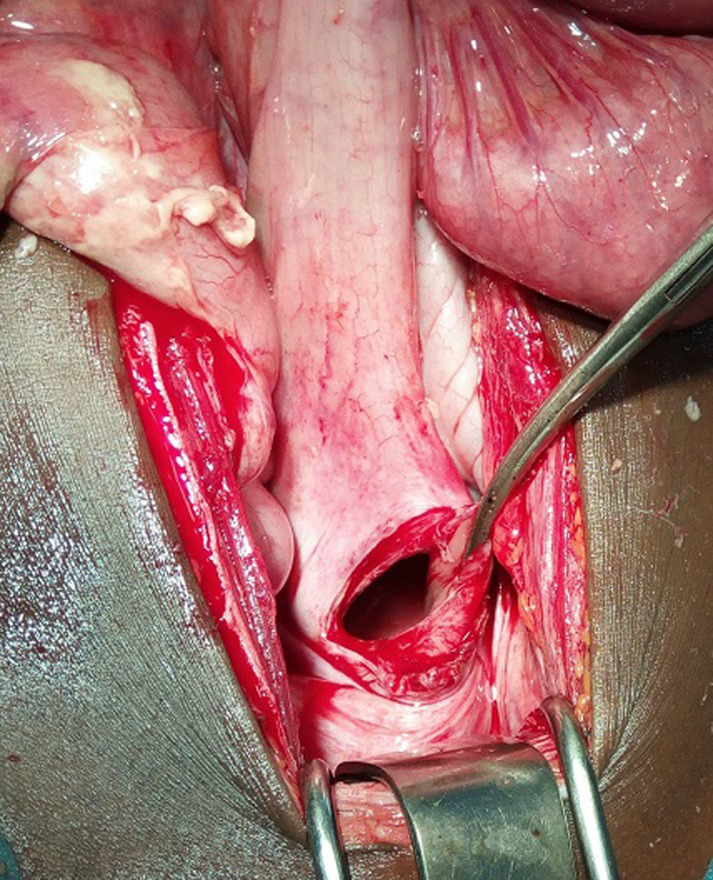
perforation rectale

Il s'agit d'un adolescent âgé de 11 ans, admis au service des urgences du Centre Hospitalier National de Ourossogui (Nord du Sénégal) pour une issue des anses grêles à travers l'anus évoluant depuis 2 heures. Il s'agissait d'un traumatisme direct ouvert du rectum à travers l'anus par chute avec réception sur un morceau de bois pointu. L'examen clinique avait retrouvé un bon état général, une issue, par l'anus, d'environ 25 cm d'intestin grêle viable (Figure 1) et un abdomen sensible dans son ensemble. Le reste de l'examen clinique était sans particularité. Le bilan biologique pré-opératoire était normal et aucune imagerie n'était réalisée. Après une réanimation, l'exploration chirurgicale par laparotomie médiane sous ombilicale avait mis en évidence un liquide séro-hématique (300 ml) et une issue d'environ 60 cm d'anse grêle viable mais inflammatoire à travers une brèche d'environ 5 cm de la paroi antérieure du rectum (Figure 2). Une réduction des anses grêles éviscérées par traction douce, une réparation de la paroi rectale par des points séparés, une toilette et un drainage ont été réalisés. Le patient a été sous antibiotique à large spectre. Les suites opératoires étaient simples avec une reprise du transit à J2 post-opératoire. La sortie a été autorisée à J7 post-opératoire. Après un recul d'un mois, l'enfant a été revue en consultation et il était libre de tout symptôme.

## Discussion

L'éviscération transanale du grêle est un phénomène rare chez l'enfant [1]. Elle est plus rencontrée chez les patients âgés. C'est la raison pour laquelle ses facteurs étiologiques sont mieux documentés chez les adultes que chez les enfants [2]. Plus de 100 cas de ce type ont été rapportés dans la littérature, mais seulement une vingtaine de cas sont décrits chez les enfants [2]. Les causes d'éviscération transanale de l'intestin grêle chez l'enfant sont dominées par les traumatismes abdominaux contondants [1-3] et les accidents d'aspiration sur bonde de piscine [2, 4, 5]. La revue de la littérature a permis de retrouver un certain nombre de cas d'éviscération transanale de l'intestin grêle chez l'enfant par empalement [6] suite à une chute sur un objet tranchant [6, 7] et une blessure auto-infligée [8]. Les lésions associées intra et/ou extra-abdominales sont souvent sévères et peuvent rendre le tableau clinique plus dramatique [2, 9, 10]. C'est la raison pour laquelle un bilan pré-opératoire complet est de la plus haute importance. Un interrogatoire détaillé permettra de préciser l'identité de l'objet ainsi que le trajet et la direction de la pénétration, des notions d'hématochézie, d'hématurie, et douleur abdominale.

Un bilan paraclinique telles que l'ano-rectoscopie, la vaginoscopie, et la cystoscopie sous anesthésie générale devraient être effectuées s'il y a un soupçon de blessure pénétrante pour évaluer l'étendue de la blessure [6]. Aucun bilan endoscopique n'a été réalisé chez notre patient du fait de leur indisponibilité dans cet hôpital. L'exploration chirurgicale permet de mieux apprécier les lésions abdominales. Au terme de ce bilan lésionnel, on peut classer les blessures d'empalement ano-rectal, en se basant d'abord sur le trajet de l'empalement comme étant soit trans-anal ou trans-périnéale et puis en subdivisant en lésions intrapéritonéale ou extrapéritonéale [6, 11, 12]. Chez notre patient, l'empalement était transanal et avait entraîné une perforation intrapéritonéale du rectum. Le traitement de cette condition suit des principes de base [2]. L'intestin grêle doit être réduit doucement et s'il n'est pas viable, il doit être réséqué [2]. Par conséquent, si la viabilité de l'intestin grêle est discutable, il peut être conservé mais un deuxième coup de fil est conseillé [2].

Dans notre cas, il y avait une souffrance intestinale donc nous l'avions préservé. La déchirure du rectum peut être réparée ou réséquée [6, 13]. Une fermeture primitive de la plaie rectale est réalisée si les lésions sont limitées et en l'absence de contamination fécale intra-péritonéale. Dans le cas contraire, la suture rectale peut être protégée par une colostomie de proche amont. Habituellement il n'existe pas de contamination fécale de la cavité péritonéale en raison du colmatage de la plaie rectale par les anses éviscérées [1, 6, 9]. Cela explique que certains préfèrent la fermeture de la plaie rectale sans dérivation des matières [2] alors que d'autres optent pour une fermeture associée à une colostomie de proche amont [6]. Pour notre part, nous avions choisi de faire une suture rectale sans colostomie de protection. Le pronostic des lésions transanales par empalement est généralement bon, lorsque le principe du traitement est respecté [6, 14], comme c'est le cas chez notre enfant.

## Conclusion

L'éviscération transanale de l'intestin grêle est rare chez l'enfant et le mécanisme par empalement reste exceptionnel. Les explorations radiologiques ne doivent pas retarder la chirurgie surtout dans le contexte africain où l'accès à ces explorations est parfois difficile. La conservation de l'intestin grêle éviscéré doit être privilégiée en l'absence de perforation ou de nécrose.
